# Evaluation of the postal service for referral of specimen of drug resistance tuberculosis in Amhara region, Ethiopia; mixed method

**DOI:** 10.4314/ahs.v21i2.17

**Published:** 2021-06

**Authors:** Gebremedhin Berhe Gebregergs, Mulusew Alemneh Sinishaw, Melashu Balew Shiferaw, Tenagnework Antife, Melkie Assefa, Daniel Fiseha, Eveline Klinkenberg

**Affiliations:** 1 School of Public Health, College of Health Sciences, Mekelle University, Mekelle, Ethiopia; 2 Amhara Public Health Institute, Bahir Dar, Ethiopia; 3 Research and Technology Transfer Core Process, Amhara Regional Health Bureau, Bahir Dar, Ethiopia; 4 Private Health Sector Program (PHSP), Abt Associates Inc, Addis Ababa, Ethiopia; 5 KNCV Tuberculosis Foundation/USAID Challenge TB, Addis Ababa, Ethiopia; 6 KNCV Tuberculosis Foundation, The Hague, the Netherlands; 7 Department of Global Health, Amsterdam Institute for Global Health and Development, Academic Medical Center, Amsterdam, the Netherlands

**Keywords:** Postal service, specimen referral, turnaround time, drug resistance tuberculosis

## Abstract

**Background:**

In Ethiopia, specimens of presumptive drug resistant tuberculosis cases are transported by courier system from district sample collection centers to reference laboratories. It is essential to track the effectiveness of the referral system and identify challenges in order to take timely and appropriate actions. We assessed turnaround time and quality of specimens, and explored challenges of the specimen referral system in Amhara region, Ethiopia, 2017.

**Methods:**

With mixed methods, we retrospectively examined 385 randomly selected presumptive drug resistance TB specimens, and interviewed 53 purposively selected key informants from laboratories and post offices. We calculated median TAT and proportion of acceptable quality. We analyzed qualitative data thematically.

**Results:**

Of the 385 specimens, 94.5% (364/385) had acceptable quality at arrival in the reference laboratories. All the 364 specimens had result. Three - fourth (76.1%) of results were dispatched to the referring health facilities within the recommended turnaround time. Ineffective communication and lack of feedback among institutions were mentioned as challenges.

**Conclusion:**

The postal service was effective in keeping quality and majority of test results were timely delivered. Yet, there were operational challenges. Therefore, effective communication, using dedicated vehicle for specimen shipment and awareness creation on specimen collection and handling are recommended.

## Introduction

Despite the rapid scale-up in recent years, rapid molecular tests like GeneXpert for the diagnosis of tuberculosis (TB) are not yet widely available in every health facility. Thus, facilities capable of performing sensitive TB diagnostic and rapid drug resistance TB tests are highly centralized^1^. To increase access to advanced diagnostic tests, investing in a comprehensive sample transportation network is essential^2^.

In Ethiopia, samples of presumptive drug resistance TB cases from Directly Observed Treatment Shortcourse (DOTS) centers are transported using the national postal services to reference laboratories to perform culture and/or Drug Susceptibility Test (DST) for diagnosis and monitoring of treatment follow up^3^. A network of District Sample Collection Centers (DSCC) for specimen referral was designed based on geographic proximity from referral centers. Health professionals bring the specimens from remote health facilities to the DSCCs. Then, from there, specimens are transported using the postal service, the current established courier system, to DST centers and regional laboratories. The transportation is usually made by car, and sometimes by motorcycle. Regional laboratories monitor the system in terms of the nuber of specimens tested, delivery of test results and turnaround time^3^, ^4^.

In Rwanda, public transportation was considered a model of sample transportation within the national laboratory network. However, it does not provide a solution to the safety and liability concerns because they had transported samples with other cargo and passengers^5^. According to the WHO guideline, packaging of infectious materials for transport must adhere to standards and be designed to minimize the potential for damage during transportation and it must ensure the integrity of the materials^6^. Inadequate sample preservation during transit can result in multiple diagnostic and therapeutic issues: culture contamination; invalid test results; the need for repeated patient sampling (with inherent delays to revisit patients/collection sites and transport each sample); and, consequently, significant delays in initiating effective treatment^7^.

The efficacy of the laboratory smears examination, nucleic acid amplification assays, culture, and MTB identification procedures, as well as drug susceptibility testing from clinical specimens, depends upon the proper collection and timely transport of specimens^8^.

According to the national GeneXpert MTB/RIF Assay Guideline, Xpert MTB/RIF test result and LPA for specimens from outside of the testing site (referral samples) should be delivered within five days (TAT) after the patient submits the specimen (4). For culture samples, the set turnaround time (TAT) is 64 days using solid media and 30 days when using liquid media^9^.

In Amhara region, Ethiopia, there are 820 health centers, 19 hospitals and two regional laboratories. One hundred fifty DSCCs collect sputum specimens of presumptive drug resistance TB cases from remote health centers, also called referring laboratories. There are 17 DST centers and one regional laboratory that perform drug resistance TB (DR-TB) diagnosis10. Specimens for DR-TB testing are transported using the postal service to DST centers (for Gene Xpert and LPA) and regional laboratories (for culture) accordingly. The process consists of several steps: DSCCs collect and pack specimens from presumptive drug resistance TB cases. They put a tracking log on the package. An individual from the post office picks up after being informed by the DSCC and at pick up s/he puts barcode including schedule and location on the package. The post office ships 3–5 triple packed* specimens on average to reference laboratories. On arrival at the reference laboratory, specimens are checked for quality. When specimens are rejected for being of poor quality, the staff in the referring laboratory is informed through the specimen rejection form via the postal system. Then he collects a new specimen from the patient and sends it using the postal system.

**Fig 1:**
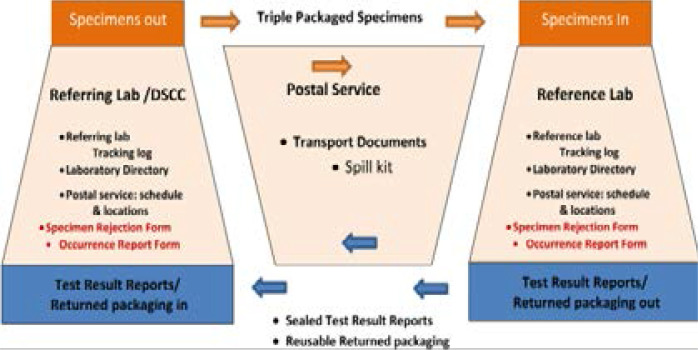
Specimen referral system in Ethiopia, 2017 *Source: Specimen referral system training module of the African Center for Integrated Laboratory training* 11

After the diagnostic tests are performed, result reports are packed and prepared for transportation. The post office man comes based on the schedule and returns the packed results out to their specific locations (Fig 1). *Triple package= container with inner primary and secondary watertight packaging and rigid outer packaging that protects the first and second box from physical damage and water.

Although the postal drug resistance TB specimen referral system is implemented in the Amhara region since 2013, adherence to the set turn-around time has never been evaluated. Moreover, anecdotal reports are indicating there are some inconveniences and miscommunications about specimen delivery. It is essential to track the effectiveness and efficiency of the referral system to identify challenges to take timely and appropriate actions. Hence, the present study was developed to evaluate whether the postal service delivers acceptable quality specimens and transmit back test results within the set time frame within the Amhara region, Ethiopia.

## Methods

We undertook a retrospective study to evaluate the postal service for specimen referral of drug resistance tuberculosis in Amhara region from November 20 to December 31, 2016. We also qualitatively explored the anecdotally reported challenges of the specimen referral system. We investigated 385 randomly selected presumptive drug resistance TB specimens, and interviewed 53 purposively selected key informants from the post office, DSCC and Diagnostic sites.

Using the single population proportion formula, we calculated the target sample size using Epi Info version 3.5.1. We hypothesized 50% of samples would have acceptable quality, 95% confidence interval and 5% margin of error. For this study, we included 14 reference laboratories that provide a diagnostic test for drug-resistant tuberculosis. In each diagnostic site, specimens were selected using simple random sampling technique and computer-generated random numbers. We had included 14 Key informants (KIs) from diagnostic centers, 13 KIs from post office and 26 KIs from DCCs. Key informants were purposively selected from each service level based on their involvement in the referral system. Specimens were followed starting from the date of collection to their arrival time at the reference site. Then, results were followed until they were dispatched to the referring sites. In doing so, records (drug resistance TB specimen registration log, postal package and receipt form) were reviewed using a structured checklist to determine the proportion of acceptable quality specimens delivered through postal service and their respective TATs. Besides, key informants were interviewed using a structured interview guide to inquire about the existing challenges of the specimen referral using the postal system.

Data collection was done by laboratory personnel from hospitals. The investigators selected, trained and supervised the data collectors. Each questionnaire was reviewed and checked for accuracy and completeness by the study team before being entered.

We considered a sputum specimen of acceptable quality if it was held with triple packaging at 2–8 °C and arrived within five days of collection; it was none- bloody and had no food lefts. Such data is found at the reception house of the diagnostic center. We defined TAT as the time between submitting a specimen to the reference laboratory and receiving test the results back at the referring laboratory. Set time for this is 5 days for GeneXpert, 64 days for solid culture and 30 days for liquid culture. In this study, we used the phrase ‘non-analytical phase’ to indicate the time that specimens stayed in the pathway/courier system, and the analytical phase to the time specimens stayed at the diagnostic center.

### Data analysis

Each questionnaire was reviewed and checked for accuracy and completeness by the study team before being entered. Epi-Info™ version 3.5.1 (Centers for Disease Control and Prevention, Atlanta, GA, USA) was used for data entry. SPSS version 20 (Statistical Package for the Social Sciences, Chicago, IL, USA) statistical package was used for data analysis. Descriptive statistics were used to determine the proportion of specimens with acceptable quality. Median TAT was calculated as the data distribution was positively skewed. Wilcoxon Signed Rank test was performed to compare the median TAT between analytical and non-analytic phases. We considered a p-value < 0.05 as statistically significant.

For the qualitative part, we thematically analyzed the data. The team started analysis during data collection. Field notes were reviewed and translated into English daily. All the field notes, transcripts and translations were read repeatedly till the investigators became familiar with them. Codes were developed based on the original terms used by participants, followed by identifying the most frequently observed categories and development of themes.

### Ethical considerations

The study was reviewed and approved by the Regional Ethical Review committee of the Amhara Regional Health Bureau. A letter of permission was obtained from the head of each post office, diagnostic sites, and DSCCs. Verbal consent was obtained from each key informant. Interviews were conducted in a private area. The right of withdrawal from the study was assured at any time during data collection. All the data were coded and only accessible to the research team to assure confidentiality. Data were we de-identified and de-linked.

## Results

### Quantitative findings

#### Referral pattern and quality of drug resistance TB specimens

We investigated a total of 385 presumptive drug resistance TB specimens that were transported by the postal system. Of the total specimens, 356 (92.5%) were transported for DST (X-pert and LPA). The majority of specimens, 348 (90.4%), were transported to hospitals for diagnosis. Of the total specimens, 364 (94.5 % with 95% CI: 91.9% to 96.5%) were received at the reference laboratories with acceptable quality. The rest (5.5 %) was rejected for different reasons: 0.8% due to being bloody, 1.0% arrived after 5 days of collection, 0.8 % because out of the range temperature, while three (0.8%) specimens were mixed with food remnant. For 9 specimens (2.1%) no information was available on why they were rejected.

Out of 356 samples sent for DST, 335 (94.1%) had acceptable quality. All of the samples sent for both culture and DST had acceptable quality. All the 364 good quality specimens were processed and had a result (table 1).

**Table 1:** Referral pattern and quality of drug resistance TB specimens in Amhara region, 2017

Variable	Category	Frequency	Percentage
**Diagnostic facility**	Health center	8	2.1
	Hospital	348	90.4
	Regional Laboratory	29	7.5
**Reason for referral of specimen**	DST(Xpert/LPA)	356	92.5
	Both DST and culture	29	7.5
**Acceptable quality**	Yes	364	94.5
	No	21	5.5
**Quality indicators**			
**Triple package**	Yes	382	99.2
	No	3	0.8
**Temperature within range**	Yes	382	99.2
	No	3	0.8
**Arrival within 5 days of** **collection**	Yes	381	99.0
	No	4	1.0

* DST=Drug Susceptibility Test; LPA=Line Probe Assay

### Turnaround time of drug resistance TB specimens

Over three quarters (76.1%) of results were dispatched to referring health facilities within the recommended TAT. The median TAT of specimens sent for Xpert/LPA was one day (Interquartile Range (IQR) = 4 days). The median TAT of specimens sent for culture and DST was 129.0 days (IQR=59.5 day). Based on the recommended TAT (5 days for Xpert/LPA and 64 days for culture), 277 (82.7%) specimens sent for Xpert/LPA were in time, but all, 29 (100%), specimens sent for both DST and culture were delayed (Table 2).

**Table 2:** TAT status of drug resistance TB specimens sent for DST (Xpert/LPA) and culture in Amhara region, 2017

Reason for referring the specimen	Median(IQR) in days	TAT-delayβ	
		Yes	No
DST(**n=335**)	1(4.0)	58(17.3%)	277 (82.7%)
Both Culture and DST(**n=29**)	129 (59.5)	29 (100.0%)	0 (0.0%)
Total (n=364)	1(5.0)	87(23.9%)	277 (76.1%)

* DST=Drug Susceptibility Test; TAT=Turnaround Time

β Results arrived outside the time set of the different test

There was a significant difference (p<0.001) in the number of days that specimens stay on the pathway (none analytical) and analytical phase. Looking in detail at the delayed specimens, they stayed a median of six days in the reference lab before being transported and subsequently a further median of six days in the courier system before getting back to the referring laboratories/DSCC (table 3).

**Table 3:** Wilcoxon Signed-Rank for TAT of delayed presumptive drug resistance TB specimens in Analytical and postal system (none- analytical period) in Amhara region, 2017

Variable	Wilcoxon Signed Rank Test for TAT				
For delayed specimens	N	Median in days	IQR in Days	Median difference	P-value
TAT at analytical phase	87	6	77.0	0	<0.001
TAT at none analytical phase	87	6	9.0		

TAT- Turnaround Time; IQR=Interquartile Range

**Fig 2:**
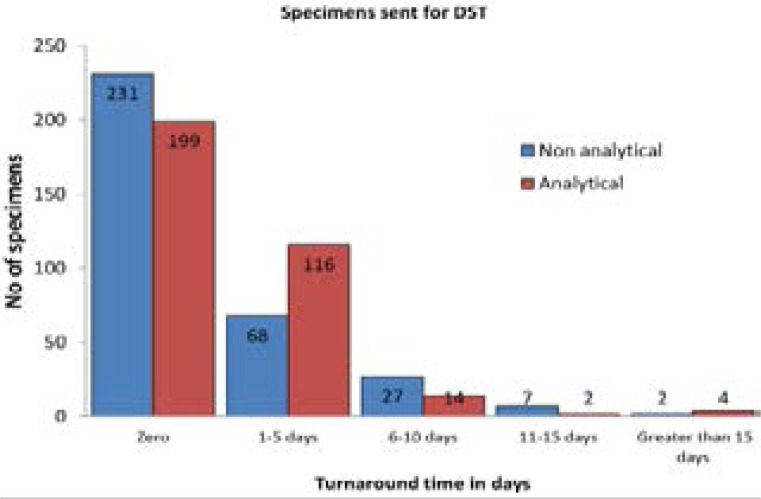
Turnaround time (TAT) of presumptive drug resistance TB specimens(N= 335) sent for DST in analytical and none-analytical phases in Amhara region, 2017

Regarding specimens sent for DST, the TAT was zero days for 231(59.0 %) and 199 (59.4%) specimens at the none-analytical and analytical phases, respectively. However, the TAT was greater than 15 days for two (0.6%) specimens at the non-analytical phase (postal system) and four (1.2%) specimens at the analytical phase (reference laboratory); Fig 2.

**Fig 3:**
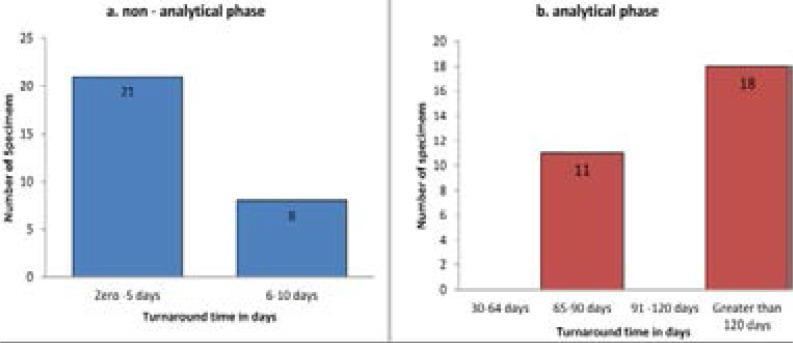
Turnaround time of presumptive drug resistance TB specimens sent for both culture and DST (N= 29) in none-analytical and analytical phase in Amhara region, 2017

For the none-analytical phase (courier system), the TAT was zero up to five days for 21 (72.4 %) specimens, however processing of the samples at reference laboratories, took more than 120 days for 18 (62. %) specimens sent for culture and DST (Fig 3).

During the investigation of TAT in the health facility where specimens were referred to, all (29) samples sent to Bahir Dar regional laboratory and 9 (69.2%) samples to Debre-Tabor were delayed. All samples (34 and 8) sent to Kemissie and Metema hospitals were in time (S1 table).

### Qualitative findings

**Theme I:** Reporting mechanism

Of the 53 KIIs, 50 participants mentioned that there was no formal reporting system between post office and diagnostic centers, and between the post office and referring health facilities. Participants mentioned that the post office personnel that came from DSCSs took the test results when they bring specimens to be diagnosed. For example, “Sometimes, we called to the nearby post office when results are ready and if they are late based on the schedule.” (Male participant, diagnostic center)

**Theme II:** Merits of postal service in specimen referral The use of postal service for transportation of presumptive drug resistance TB specimens has created a lot of benefits to stakeholders involved in the system, as mentioned below. However, few key informants reported that they observed no merit at all.

**Timely delivery of test results:** More than two-third in-depth interview participants revealed that test results can be delivered on time when transported by the system as there is a dedicated person at the post office in charge of that. Besides this, in some DSCC, post offices have a motorcycle to transport the specimen. This has solved the problem of specimen transport since most health facilities have no vehicles for this purpose. A 29-year-old man form the post office explained: “*I feel it is my national and social responsibility to timely deliver test results to health facilities and finally contribute to the prompt treatment for patients*.”

**Diagnostic access:** Some key informants mentioned that, these days, TB patients have saved their energy and time. . They do not have to travel to other facilities for diagnosis as their specimens are sent by the courier system. This saves the patient's money and time; above all, patients are getting timely treatment. Moreover, patients' results are kept confidential, as they are sealed under the envelope.

Almost all participants described that referring health facilities can optimally utilize laboratory personnel as they are in place every day to work their routine activities and do not have to take time to transport samples. Source of income to post office: Apart from the advantages for the patient and health facilities in general, participants from the post office noted an increment of income for the post office. It has also created inter-sectorial collaboration between the health bureau and post office.

**Theme III:** Challenges of specimen referral mechanism

During the exploration of barriers encountered in the specimen referral system, more than 50 percent of the key informants mentioned the existence of various challenges with the postal service.

There was no good collaboration/cooperation between the post office and health professionals. This was manifested by the absence of a formal reporting system, no forum of discussion among parties. In the postal envelope, sometimes even proper address where specimens came from is missed.

Since there is no established system to monitor the arrival of results at DSCC, there are times when test results are lost.

Currently, majority of specimens are transported using public transport as post offices do not have a dedicated vehicle for sample transportation. This can expose the passengers to infection. Besides, a key informant from DSCC mentioned that specimens are not transported in the appropriate cold temperature when the post office is using public vehicles. Samples and other goods of passenger are transported together.

Six participants from diagnostic centers and nine participants from DSCCs noted that results were not timely delivered to specimen referring sites. Even people did not give full attention to it, and there is no follow up if results do not arrive.

## Discussion

Proper collection and transport of clinical specimens are critical for the identification of drug resistance tuberculosis^12^. It ensures specimen integrity for an accurate diagnosis. The present study evaluated the effectiveness (in terms of TAT and quality of specimens transported) of the drug-resistant tuberculosis specimen referral system through postal service in the Amhara region, Ethiopia.

In this study, 94.5 % of specimens that were transported by post office arrived with acceptable quality and all of them had test results. Of the total, 76.1% arrived within the set TAT. This means that for the majority of samples, the sample transportation worked well. A study in Uganda showed a different picture with 64% of samples being transported reported to be of poor quality; 16% were not triple packaged, 8% were <3 mls, 30% were not delivered on time, and 39% were salivary in appearance^13^. The use of different quality indicators in this study could partly explain the variation.

Although three-quarters of results were dispatched within the set TAT, it was observed that there was a significant delay during the analytical phase at the diagnostic sites before samples were collected by the courier system. Diagnostic tests conducted in regional laboratories were all delayed. This may be due to three things: one, in regional laboratories two different tests (DST & culture) are commonly made for each specimen. Specimens sent for both culture and DST have a longer sample processing time; second, although the test is performed, the report may not be prepared on time, and thirdly, poor communication between the technical staff and liaison officer at the diagnostic facility could lead to results being dispatched late. Further research needs to be undertaken to ascertain the reasons for this. The observed TAT for some samples in this study was longer than the national set standard of 5 days for DST and 64 days for culture^4^,^9^. Other studies in Ethiopia reported that in health facilities supported under Public-Private Partnership (PPP), the average TAT was reduced from 7 days to 2 days in Addis Ababa and 10 days to 5 days in Amhara region. This was achieved after the PPP supported procuring of 400 standard specimen containers and the training of 586 laboratory personnel and 81 postal workers^14^. The observed difference with our study could be linked to the intervention they had instituted in the partner supported health facilities.

Timely delivery of test results and proper utilization of health personnel were mentioned among the advantages of the courier system. Due to the use of postal service for transportation, laboratory staff has more time to dedicate to the processing of samples. Besides, patients now also do not need to travel and make additional costs for transport. Similar observations are reported from Vietnam where it was reported that the specimen referral system is more convenient as laboratory staff does not need to travel to diagnostic facility carrying samples. It is safe for staff and the environment^15^.

In this study, despite the positive findings, several challenges were identified with regards to sample transportation by the postal service, i.e. I) Use of public transport: specimens are transported using public transport; meanwhile the desired low-temperature range may not be maintained. According to the guild line for specimen referral system, specimens for TB Culture and DST should be transport at 2 to 8°C 16. Although public vehicle is an option to transport samples, it is crucial to prevent damage and cross-contamination, and maintain transportation conditions^17^. However, public vehicles might not have enough space to store samples until the courier arrives; because samples are shipped together with other goods of passengers. Besides, packaging of infectious materials for transport must adhere to standards and be designed to minimize the potential for damage during transportation and it must ensure the integrity of the materials^6^. II) Weak communication system (between post office personnel and laboratory staff) and lack of systematic feedback: although the drug-resistant TB specimen referral system through postal service has been implemented widely in the Amhara region, there is no established system to check whether results have arrived back at the DSCC or not. This has resulted in loss of some reports before getting back to peripheral health facilities. A similar problem was observed in Peru where coordination of activities received inadequate priority among program planners in streaming specimen transport^18^. This may implicate weak intersectional collaboration in our setting, Ethiopia.

Effective communication is necessary when many peripheral health facilities send sample to a single referral diagnostic center^19^. TB control requires prompt, complete, and accurate communication among the laboratory systems, TB-control programs, and health-care providers^20^.

Currently, the postal service for sample transportation is available in all districts of the region. It is funded by the Federal Ministry of Health through the Ethiopian Public health Institute. Although the system is working well overall, it can benefit from the administrative commitment and systematic monitoring of the postal service and laboratory performance. This needs an active engagement of the post office personnel in review meetings with the health sector.

## Limitation

Data were collected from existing records that may have been inaccurate. There were some incomplete data; for example reasons of rejection were not documented for some specimens.

Besides, the short observation period could be a limitation to our study.

## Conclusion

We have shown that the postal system was effective in delivering acceptable quality specimens within the recommended turnaround time; nevertheless, several challenges were identified. Intensive awareness creation on specimen collection and handling is needed for laboratory personnel and individuals in the post office as well as systematic monitoring of results delivery. The transport system would benefit from dedicated vehicles across its structure for specimen transportation and yet its cost-benefit analysis needs further research. There need to be a common understanding among all stakeholders by creating a forum of discussion and sensitization program.
